# Case Report and review of the literature: esophageal pleomorphic rhabdomyosarcoma

**DOI:** 10.3389/fonc.2025.1511957

**Published:** 2025-02-28

**Authors:** Matteo Pittacolo, Arianna Vittori, Lucia Moletta, Gianpietro Zanchettin, Elisa Sefora Pierobon, Giovanni Capovilla, Renato Salvador, Mauro Michelotto, Michele Valmasoni

**Affiliations:** ^1^ Department of Surgery, Oncology and Gastroenterology, University of Padua, Padova, Italy; ^2^ 1st Surgical Clinic, University Hospital of Padua, Padova, Italy; ^3^ Surgical Pathology Unit, University Hospital of Padua, Padova, Italy

**Keywords:** esophageal rhabdomyosarcoma, prognosis, management, metastases, esophagectomy, case report

## Abstract

**Background:**

Esophageal rhabdomyosarcoma is an exceedingly rare malignant tumor, with only three cases comprehensively documented in the literature since 1995. Due to its rarity, there is limited information on the epidemiology and diagnosis of this disease, and no standardized treatment protocols have been established. As a result, both the recognition and management of esophageal rhabdomyosarcoma pose significant challenges. The present case report provides valuable insight into the clinical approach to this rare tumor, highlighting the need for further research and investigation to develop more effective diagnostic tools and therapeutic strategies.

**Case Presentation:**

We described the case of a 77-year-old male who presented with dyspepsia and anemia, leading to the discovery of an esophageal lesion. At the index endoscopy, histological findings were consistent with esophageal adenocarcinoma. Consequently, the patient was treated with neoadjuvant chemotherapy and Ivor Lewis esophagectomy. Interestingly, on pathological examination the lesion was identified as a pleomorphic rhabdomyosarcoma of the esophagus. Postoperatively, the patient received adjuvant chemotherapy. Subsequently, a subcutaneous metastatic lesion on his right shoulder was treated with a combination of radiotherapy and surgical excision. The patients died twenty-six months after the initial diagnosis.

**Conclusions:**

Our case represents one of the few reported instances of esophageal rhabdomyosarcoma, a highly rare and aggressive malignancy, and provides valuable insights into the challenges of diagnosing and managing this disease. Moreover, this is one of the first cases of esophageal rhabdomyosarcoma followed up for more than 24 months. However, given the paucity of data on esophageal rhabdomyosarcoma, there remains a significant unmet need for more comprehensive studies to establish standardized diagnostic and therapeutic protocols.

## Introduction

The two most common histological types of esophageal cancer are adenocarcinoma and squamous cell carcinoma, while esophageal sarcomas are exceedingly rare, accounting for only 0.2% of patients undergoing esophagectomy for cancer (1). Among these, primary esophageal rhabdomyosarcoma (RMS) is an especially rare subtype, with fewer than 20 cases mentioned in the literature (2). Due to its rarity, there is no established management protocol for this tumor. Additionally, esophageal RMS is often misdiagnosed as esophageal carcinoma, as distinguishing between these tumors under a light microscope is difficult due to the presence of undifferentiated cells, which complicates diagnosis.

In this report, we present the case of an esophageal neoplasm that, only upon pathological examination, was identified as a pleomorphic rhabdomyosarcoma. The patient was managed at a specialized academic referral center for esophageal diseases. Moreover, a narrative literature review was conducted to examine current evidence on esophageal rhabdomyosarcoma.

## Case presentation

A 77-year-old Caucasian male was referred to our center due to a three-month history of dyspepsia, with no reported dysphagia. The patient was 176 cm tall, weighed 73 kg, giving him a body mass index (BMI) of 25.6 kg/m², and had a Karnofsky Performance Status (KPS) score of 80. He worked as a real estate businessman and had a significant smoking history, having been a heavy smoker until five years prior to presentation. He also consumed alcohol occasionally. There was no known family history of malignancies. His past medical history was notable for a thyroidectomy for thyroid cancer, and he was on thyroid hormone replacement therapy.

Eight months before diagnosis, the patient developed sideropenic anemia, which had been managed with recurrent blood transfusions. Additionally, he had a positive fecal occult blood test three months prior to our evaluation. On physical examination, no abnormalities were detected. Blood tests were unremarkable, and tumor markers, including carcinoembryonic antigen (CEA) and carbohydrate antigen 19-9 (CA19-9), were within normal limits. Esophagogastroduodenoscopy (EGD) revealed a stenosing lesion located from 1 cm above to 2 cm below the cardia, which appeared ulcerated and prone to easy bleeding.

**Figure 1:**
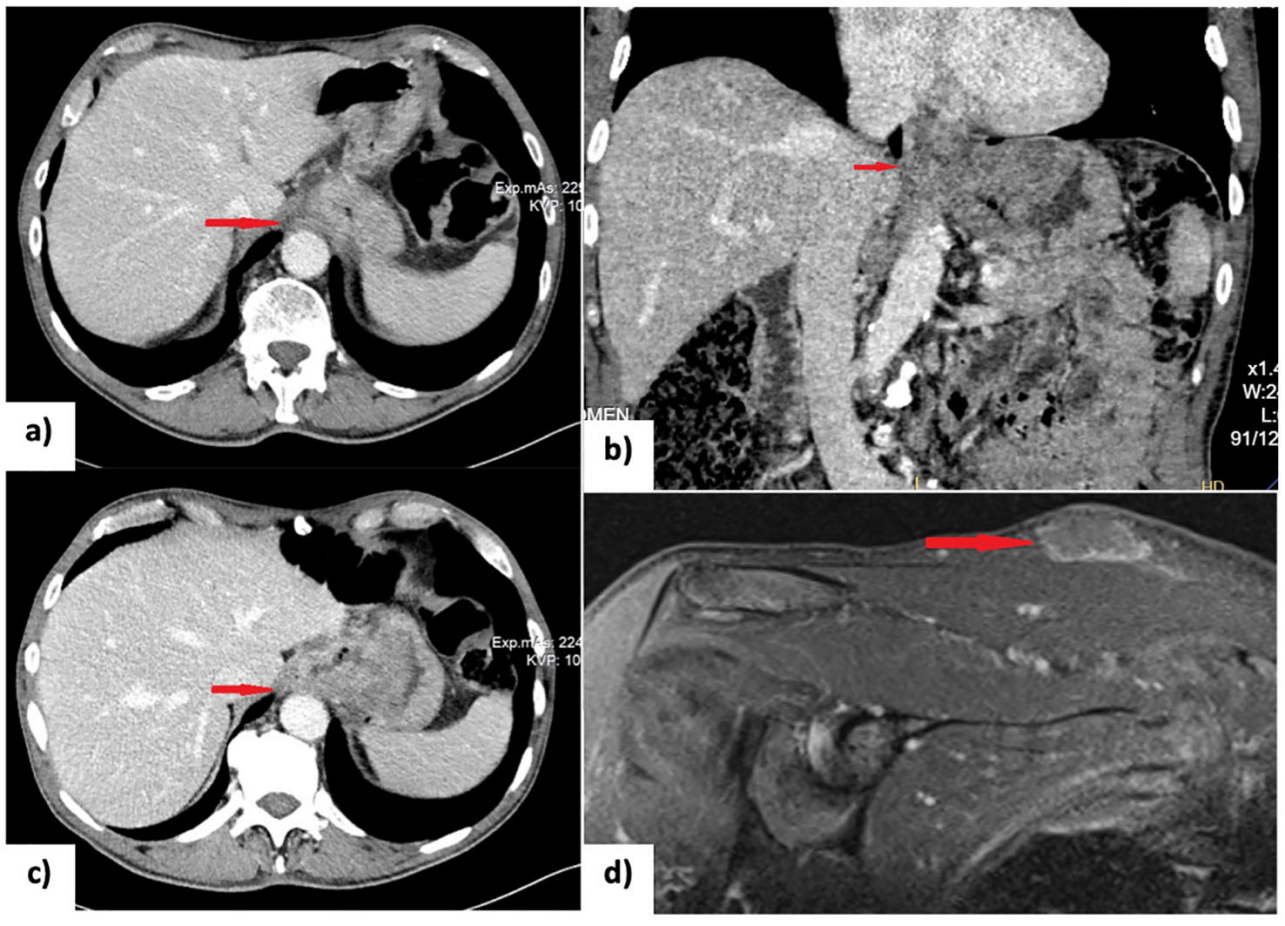
**(A, B)** CT scan at diagnosis showing the gross circumferential stenosing thickening of the cardia; **(C)** Restaging CT scan after 8 cycles of chemotherapy (XELOX regimen) showing a partial response; **(D)** Short tau inversion recovery (STIR) MRI scan detecting a subcutaneous lesion on the right shoulder suspected to be a rhabdomyosarcoma metastasis. EGD, esophagogastroduodenoscopy; NACT, neoadjuvant chemotherapy; PET-CT, positron emission tomography and computed tomography; US, ultrasonography.

Histopathological analysis posed a diagnosis of esophageal adenocarcinoma, with immunohistochemical staining showing CK7(+), AE1/AE2(+), and HER2(-). A computed tomography (CT) scan showed an enlarged and hypotonic thoracic esophagus, with a prominent circumferential stenosing thickening at the cardia invading the right crus of the diaphragm (**Figures 1A, B**). The lesion extended approximately 6 cm in length, with a maximum thickness of 2 cm, and exhibited solid and heterogeneous density. Additionally, the CT scan showed thickening of the gastric fundus wall, which appeared hypervascularized. Various lymph nodes were identified: one anterior to the pancreatic tail, another, measuring 5 cm in its long axis, located along the lesser curvature of the stomach, and a few confluent lymph nodes with central colliquative necrosis in the lumbar retroperitoneum. According to the 8th edition of the AJCC staging system, the tumor was staged as cT4N1M1. The presence of a retroperitoneal lymph node metastases identified the tumor as oligometastatic (M1), while metastases to two regional lymph nodes, one at the pancreatic tail and another along the lesser curvature of the stomach, accounted for the N1 classification. Finally, involvement of the right crus designated the tumor as T4.

Following a multidisciplinary team discussion, the patient was started on neoadjuvant chemotherapy based on XELOX regimen (intravenous oxaliplatin 130 mg/m² on day 1 followed by oral capecitabine 1,000 mg/m² twice daily from day 1 to day 14 included, administered every 21 days for 8 cycles). He tolerated the treatment well, with no reported chemotherapy-related toxicities. Restaging, which included EGD, CT scan, and 18-fluorodeoxyglucose positron emission tomography (PET/CT) scan, demonstrated a partial response to treatment according to RECIST criteria (**Figure 1C**) (3). The esophageal lesion was smaller, and there was no longer any evidence of the previously identified pathological retroperitoneal lymph nodes.

Consequently, a minimally invasive Ivor Lewis esophagectomy was performed without any intraoperative complications. The procedure included a laparoscopic phase for stomach mobilization and conduit creation, followed by video-assisted thoracic surgery through a right thoracic approach for esophageal resection and reconstruction using the gastric conduit (4). The esophagogastric anastomosis was performed in the thoracic cavity, above the arch of the azygos vein. This two-stage procedure allowed for extensive oncological resection and thorough lymphadenectomy, including both abdominal and mediastinal lymph nodes. The postoperative recovery was uneventful. The patient successfully resumed oral intake by postoperative day 10 and was discharged home on postoperative day 12.

**Figure 2:**
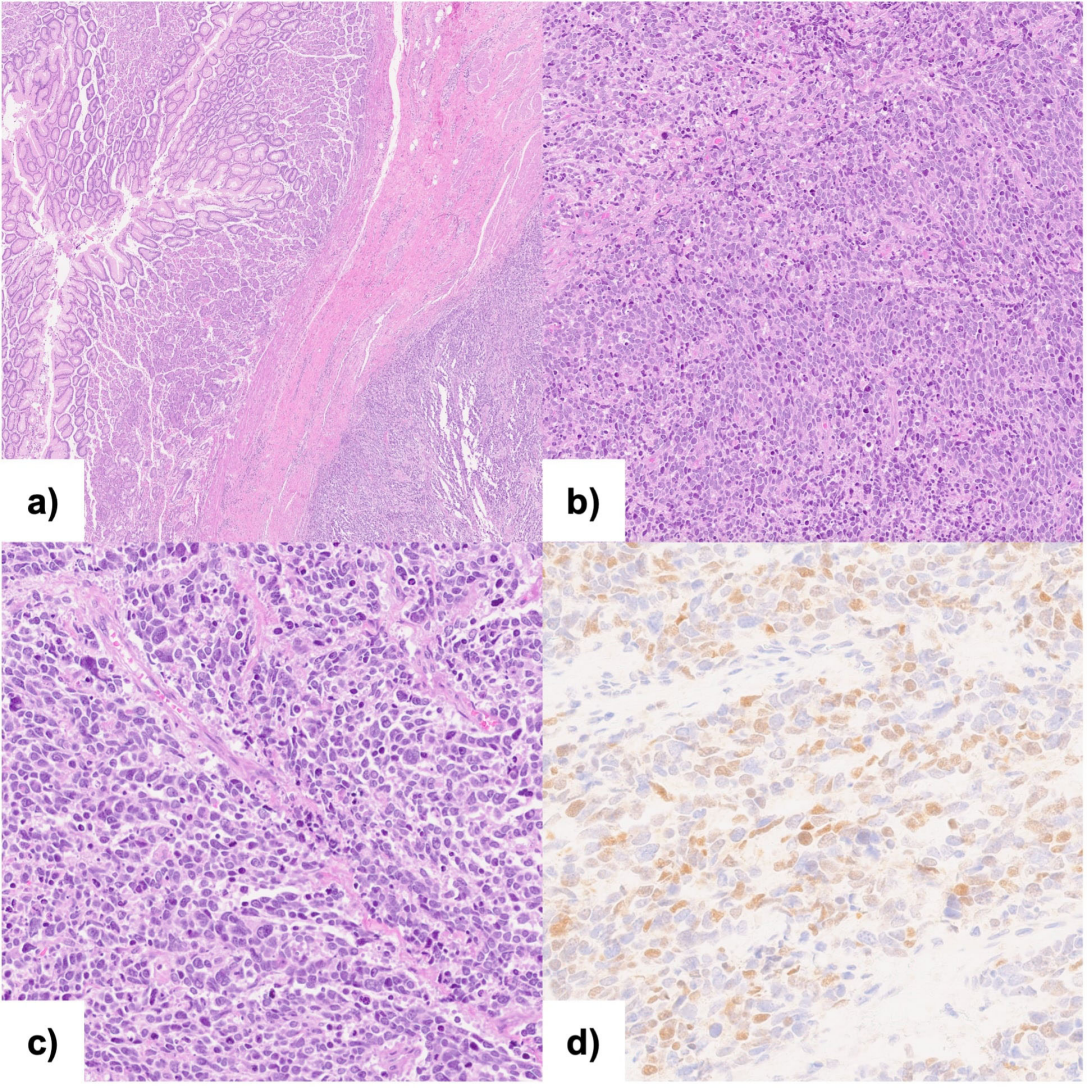
Histopathological analysis and immunohistochemical examination of the resected specimen: **(A, B)** Hematoxylin and eosin staining of the neoplasm showing pleomorphic appearance; **(C)** Hematoxylin and eosin staining of the transition zone from normal gastric mucosa to the neoplasia; **(D)** Immunohistochemical staining (40x) for Myf-4.

Although the histological findings from the endoscopic biopsy were consistent with adenocarcinoma, histopathological examination of the surgical specimen was diagnostic of esophageal sarcoma. Immunohistochemical analysis presented the following results: CDX2(-), AE1/AE3(-), S100(-), desmin(-), MYF4 (+, plurifocal), INI-1(+), TTF1(-), CD56(+), p38(+), chromogranin(-), P53(-), MLH1(++), MSH2(++), and MSH6(++), as shown in **Figure 2**. These findings were consistent with pleomorphic rhabdomyosarcoma. The esophageal lesion had a maximum diameter of 7 cm, and invaded the serosa. Of the 28 lymph nodes examined, three, located along the lesser curvature of the stomach, were positive. As a result, the tumor was classified as ypT2bN1cM0 according to the AJCC 8th edition staging system for soft tissue sarcomas of the abdomen and thoracic visceral organs. Additionally, it was graded 3 based on the FNCLCC grading system (5, 6).

Three months after discharge, the patient developed a subcutaneous lesion on his right shoulder. Ultrasound examination revealed a 36x27x9 mm inhomogeneously hypoechoic, polycyclic nodule, confined to the subcutaneous tissue, compressing but not infiltrating the underlying muscle fascia. Magnetic resonance imaging (MRI) of the right shoulder supported the suspicion of metastasis (**Figure 1D**). After a multidisciplinary discussion, the lesion on the right shoulder was surgically removed. An elliptical incision was made, and both the skin and subcutaneous tissue were excised, extending down to the muscular fascia. The surgery was followed by radiotherapy (30 Gray delivered in 10 fractions over 2 weeks) to reduce the risk of local recurrence. Histological examination of the excised specimen confirmed the diagnosis of metastatic rhabdomyosarcoma.

One month after surgery, a PET-CT scan revealed disease progression, with metastases detected in the upper mediastinum, splenorenal angle, and right kidney, along with bilateral pleural effusion. The patient was subsequently started on doxorubicin monotherapy, with six cycles planned (75 mg/m² every 21 days). Three months later, a follow-up PET-CT scan showed further disease progression, with the appearance of new contrast-enhancing lesions in the mediastinum and abdomen, as well as an increase in the size of pre-existing metastases. As a result, the chemotherapy regimen was switched to gemcitabine (1,200 mg/m2 on days 1 and 8 every 3 weeks).

Four months later, a subcutaneous lesion appeared on the right flank, and ultrasonography highly suggested metastasis. The patient was started on a third-line treatment with ifosfamide (9 g/m^2^ administered for 5 days via continuous intravenous infusion using an elastomeric pump, repeated every 21 days). One week after initiating ifosfamide therapy, the patient developed asthenia, dizziness, and balance difficulties, leading to the discontinuation of chemotherapy. A total body CT scan performed one month later revealed brain metastases. Given the patient’s declining performance status, palliative care was initiated.

**Figure 3:**
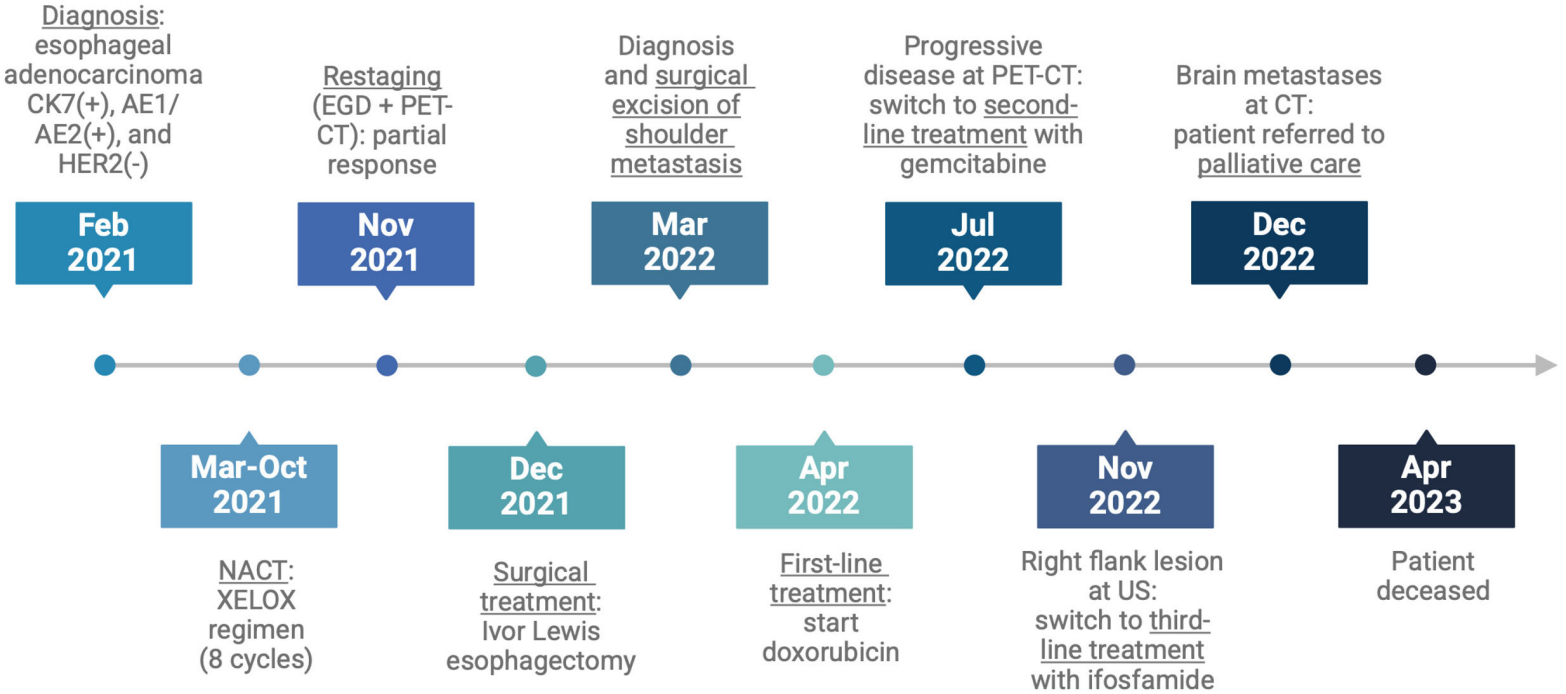
Case report timeline.

The patients died twenty-six months after the initial diagnosis. **Figure 3** shows the case report timeline.

## Literature review

Studies eligible for inclusion were those reporting treatment strategies and patient survival for esophageal rhabdomyosarcoma. All study designs were eligible for inclusion except for studies published as conference abstracts only. Studies published after 1995 were included to reflect current clinical practice. Language was restricted to English. Titles and abstracts of all identified studies were independently reviewed by two authors (M.P., A.V.), and discrepancies were resolved by a third reviewer (L.M.). After a second screening by full reading, only 3 comprehensively described cases were retrieved in the literature (**Table 1**).

**Table 1:** Articles retrieved from the literature comprehensively describing cases of esophageal rhabdomyosarcoma.

Author	Sex	Age	Sarcoma type	Primary tumor location	Primary tumor size	Treatment	Recurrence	Recurrence type	Follow-up
Batoroev et al., 2006 (7)	F	55	Pleomorphic	Lower esophagus	10 × 4 × 4 cm	Surgery + Adjuvant CT/RT	8 months	Recurrent esophageal cancer, paratracheal and peribronchial lymph node metastasis, lung metastasis	8 months (dead)
Gandhi et al. 2012 (8)	M	61	Embryonal	Middle esophagus	8.5×3.5×2.5 cm	Surgery + Adjuvant CT	9 months	Supraclavicular lymph node metastasis	9 months (alive)
Zhang et al., 2022 (2)	M	54	Embryonal	Upper esophagus	4-5 cm long	CT/RT	2 months	Multiple pulmonary metastases	8 months (dead)

CT, chemotherapy; RT, radiotherapy.

## Discussion

Esophageal rhabdomyosarcoma is an exceptionally rare form of esophageal cancer, with the first case reported by Wobbes in 1970 (9). Since then, less than 20 cases have been documented in the literature, with only three providing a comprehensive description. Rhabdomyosarcoma is primarily known as a pediatric malignancy, while in adults it accounts for less than 1% of all solid malignant tumors. The prognosis of rhabdomyosarcoma in older patients is generally poorer compared to younger individuals, reflecting differences in treatment response and disease biology across age groups (10).

For esophageal RMS, the median age at diagnosis is 58 years (2). It typically arises in the middle or lower esophagus, with common presenting symptoms including progressive dysphagia, weight loss, chest discomfort, retrosternal burning pain, nausea, and vomiting (11–13). Our 75-year-old patient presented with atypical symptoms of dyspepsia, anemia, and a positive fecal occult blood test. The prognosis for esophageal RMS is generally poor, with median survival times being short. Most reported cases in the literature have not exceeded a 10-month follow-up. In contrast, the present case reports a notable survival of over 24 months since diagnosis, which is a significant deviation from previously reported outcomes.

According to the 2020 WHO classification of soft tissue tumors, RMS are subdivided into four types: embryonal, alveolar, pleomorphic, and spindle cell/sclerosing (14). The prognosis varies significantly depending on the histological subtype. In a recent analysis, pleomorphic and alveolar rhabdomyosarcoma were associated with the poorest outcomes, with 5-year survival rates of 26.6% and 28.9%, respectively. In contrast, embryonal rhabdomyosarcoma demonstrated the most favorable prognosis, with a 5-year survival rate of 73.9% (15, 16).

At endoscopy, esophageal sarcomas typically present as polypoid and ulcerated lesions, as observed in our patient (17). Interestingly, in this case, the initial histology was consistent with adenocarcinoma, but subsequent pathological examination of the surgical specimen revealed pleomorphic rhabdomyosarcoma. Several explanations would account for this discrepancy. First, it is possible that the initial biopsies only captured the carcinomatous component of a carcinosarcoma, a scenario described in the literature (18, 19). However, squamous cell carcinoma, rather than adenocarcinoma as in our case, is the most commonly reported carcinomatous component in carcinosarcomas (20, 21). Furthermore, the pathological examination in the current case revealed no evidence of any carcinomatous components, leading us to rule out this explanation. Second, the tumor may have initially been an adenocarcinoma that later transformed into sarcoma. The potential for carcinomatous cells to undergo transformation into sarcomatous cells has been described, often attributed to stepwise gene mutations in pluripotent stem cells, with involvement of epithelial-mesenchymal transition (EMT) (22–24). However, a complete transformation into sarcoma, with no residual carcinomatous components, as observed in our case, seems unlikely, particularly within the relatively short timeframe between the initial biopsy and the pathological examination. Lastly, the most plausible explanation, considering the tumor rarity, is a misdiagnosis of rhabdomyosarcoma, a possibility that has been previously documented (15, 25). This suggests that an accurate diagnosis may require larger tissue samples, highlighting the importance of more extensive biopsies.

Immunohistochemistry is a valuable tool for establishing the diagnosis of soft tissue tumors. Rhabdomyosarcoma typically shows positive staining for myogenin, desmin, sarcomeric actin, and myoglobin, while it is usually negative for NKX2.2, CD99, CD45, cytokeratin (CK), S100, and neuron-specific enolase (NSE) (26). In our case, immunohistochemical analysis demonstrated positive staining for MYF4 (myogenin) and negative staining for S100. Although desmin is recognized as a marker for RMS, there have been reports of negative cases, as observed in the present instance (27, 28). As previously mentioned, the final diagnosis of carcinosarcoma could have explained the initial histologic diagnosis of adenocarcinoma. However, the negative AE1/AE3 staining on pathological examination allowed us to exclude the presence of a carcinomatous component, thereby ruling out the diagnosis of carcinosarcoma. While reviewing the histopathological slides from the initial biopsy would have been valuable, we were unfortunately unable to obtain them from the outside hospital where they were analyzed.

Esophagectomy remains the first-choice treatment for locoregional esophageal disease (1, 29). When a patient is unsuitable for surgery, the placement of an esophageal stent serves as an effective palliative option for alleviating dysphagia (30). Notably, there is only one documented case of endoscopic surgical treatment in the literature, which presents an intriguing possibility for new treatment avenues for patients unfit for surgery (31).

Another critical consideration in the treatment of RMS is the potential utility of compartmental surgery. Given that the completeness of resection is the most significant prognostic factor influencing treatment outcomes, the use of a compartmental resection—commonly employed in retroperitoneal sarcomas—may also improve local control and survival in cases of esophageal RMS (32). However, applying this principle to esophageal surgery presents significant challenges due to the critical proximity of vital structures. There have been no reported cases of compartmental surgery specifically for esophageal sarcomas. While partial resections of adjacent structures, such as the pleura, pericardium, or thoracic duct, may be considered as effort toward a radical surgery, these approaches would require careful evaluation of the potential risks and benefits. Nonetheless, further studies and clinical investigations are essential to assess the feasibility, safety, and outcomes of such interventions in esophageal sarcoma surgery.

The topic of systemic treatments remains controversial, with chemotherapy regimens for adult patients frequently guided by pediatric studies, and no clear consensus available in the literature. Doxorubicin is commonly used as first-line treatment in adult rhabdomyosarcomas, either as monotherapy or in combination with ifosfamide or vincristine (33, 34). In our case, however, it was discontinued due to disease progression. Recent research has indicated that combination chemotherapy, such as anthracyclines plus ifosfamide, is an effective option for managing soft tissue tumors (35). The combination of adriamycin and ifosfamide has also been described in the literature, with one study by Patricia et al. reporting disease stability of an esophageal spindle cell tumor after four cycles of treatment (36). In our patient, we were unable to assess the effects of ifosfamide, as the treatment was interrupted early due to toxicity.

In the present case, the initial diagnosis based on endoscopic biopsies was adenocarcinoma, and the neoadjuvant therapy was planned accordingly. However, as the patient was referred to an external center for oncological management and we could not retrieve any details of the consultations conducted at this outside facility, the rationale for the initial treatment with the XELOX regimen remains unclear. We hypothesize that the choice of the XELOX regimen may have been influenced by the patient’s oligometastatic status, specifically due to the involvement of retroperitoneal lymph nodes. However, according to current standards of care, FLOT is the preferred regimen for adenocarcinoma in such cases. At our center, oligometastatic patients are routinely treated with 8 cycles of FLOT. Interestingly, our patient exhibited a partial response to the XELOX regimen, particularly in the lymph nodes.

Immunotherapy is not currently considered a standard treatment option for sarcoma, although ongoing research is exploring the potential of microsatellite instability (MSI) as a biomarker for selecting patients who may benefit from these therapies (37). MSI, which reflects defects in DNA mismatch repair, has been associated with increased tumor mutational burden and may make certain tumors more responsive to immunotherapy (38). However, while our case was found to be positive for MLH1 and MSH2 expression, we lack information regarding MSI status because, at our center, this analysis is routinely performed only for adenocarcinomas and squamous cell carcinomas, rather than for sarcomas. FDA-approved immune checkpoint inhibitors targeting PD-1/PD-L1, i.e. atezolizumab and pembrolizumab, are available and have shown promise in some subsets of sarcoma (39–41). Given the emerging efficacy of these agents, PD-1/PD-L1 expression holds promise as a potential biomarker for selecting sarcoma patients who may benefit from immunotherapy, thus worth investigating in sarcoma cases. However, more data is needed to establish precise guidelines for immunotherapy use in sarcoma subtypes. Understanding the molecular landscape of these rare tumors will be crucial for optimizing patient selection and improving therapeutic outcomes.

Recent advances in the molecular pathophysiology of rhabdomyosarcomas have highlighted genetic events that correlate with tumor behavior, chemoresistance, and thus outcome, including FGFR4 mutations and RAB3IP-HMGA2 fusion transcript expression. These studies reinforce the utility of molecular profiling for identifying actionable mutations for individualized treatment development. For example, FGFR4 inhibitors may be a potentially active therapeutic approach in patients carrying such mutations. In addition, the identification of EMT- and chemoresistance-related gene signatures, including CDH1, MMP9, and LAPTM4B, provides additional information on tumor aggressiveness and potential biomarkers for prognosis (42, 43). At our institution, these investigations are not part of routine clinical practice, and as such, molecular information are not available for the present case. Integrating molecular findings into the diagnostic and therapeutic strategy for RMS can enhance the management of this rare malignancy and improve patient outcomes.

The use of radiotherapy for soft tissue sarcomas is another debated topic. In agreement with Salerno et al., radiation therapy is a viable option for patients at high risk of local recurrence and can be administered in both neoadjuvant and adjuvant settings (2, 44). Given the technical challenges associated with compartmental surgery for esophageal tumors, radiotherapy may serve as a suitable neoadjuvant treatment to mitigate the risk of local recurrence. In our case, radiotherapy was employed to manage shoulder metastasis alongside local surgical excision, resulting in excellent local control of the disease.

While the current treatment modalities offer some benefits, the prognosis for patients with esophageal RMS remains generally poor, highlighting the need for further research to establish effective management protocols. As we continue to gather data and insights from rare cases like this one, we move closer to developing a standardized approach for treating this challenging tumor type, ultimately aiming to enhance the quality of care for affected patients.

## Conclusion

Esophageal sarcomas are rare tumors associated with a poor prognosis; however, the combination of surgery, chemotherapy, and radiotherapy can significantly improve patient survival. Accurate diagnosis is crucial for implementing a targeted treatment approach, and therefore, patients should be referred to specialized centers.

Given the rarity of esophageal rhabdomyosarcoma, standard-of-care management algorithms are still lacking. Consequently, reporting individual cases is vital for the development of a common treatment strategy. Current survival data on esophageal sarcomas are limited, primarily derived from literature reviews and small series focusing on specific subtypes. There is a pressing need for additional studies exploring the pathogenesis and management of rhabdomyosarcoma.

To our knowledge, this is one of the very few comprehensively described cases of esophageal rhabdomyosarcoma reported in the literature. It shows that multimodality treatment can substantially improve survival and provides valuable insights into the management of this exceedingly rare disease.

## Data Availability

The original contributions presented in the study are included in the article/supplementary material. Further inquiries can be directed to the corresponding author.
